# Vapor-Phase Essential Oils as Antifungal Agents against *Penicillium olsonii* Causing Postharvest Cherry Tomato Rot

**DOI:** 10.3390/foods13193202

**Published:** 2024-10-09

**Authors:** Monika Mrvová, Juraj Medo, Jana Lakatošová, Zuzana Barboráková, Marcel Golian, Zuzana Mašková, Dana Tančinová

**Affiliations:** 1 Institute of Biotechnology, Faculty of Biotechnology and Food Sciences, Slovak University of Agriculture in Nitra, Tr. A. Hlinku 2, 94976 Nitra, Slovakia; monimrvova@gmail.com (M.M.); juraj.medo@uniag.sk (J.M.); zuzana.barborakova@uniag.sk (Z.B.); zuzana.maskova@uniag.sk (Z.M.); 2 AgroBioTech Research Centre, Slovak University of Agriculture in Nitra, Tr. A. Hlinku 2, 94976 Nitra, Slovakia; jana.lakatosova@uniag.sk; 3 Institute of Horticulture, Horticulture and Landscape Engineering Faculty, Slovak University of Agriculture in Nitra, Tr. A. Hlinku 2, 94976 Nitra, Slovakia; marcel.golian@uniag.sk

**Keywords:** essential oils, *Penicillium*, cherry tomatoes, antifungal activity

## Abstract

Recent reports of *P. olsonii* causing postharvest rot of cherry tomatoes emphasize the need for effective strategies to prolong fruit shelf life. This study is the first to explore the use of essential oils (EOs), recognized for their antimicrobial properties, as a potential method to prevent postharvest losses from *P. olsonii*. Antifungal activity was tested for ten EOs at a concentration of 625 μL/L using the vapor diffusion method. Thyme, wild thyme, savory, oregano, and marjoram completely inhibited fungal growth over 14 days. Thyme EO, at a minimum inhibitory concentration (MIC) of 250 μL/L, fully inhibited all strains, while oregano, wild thyme, and savory were effective at 500 μL/L. Marjoram EO showed weaker activity. The lowest IC90 values, ranging from 35.72 to 162.72 μL/L, were estimated for thyme and oregano. In cherry tomatoes, oregano EO completely halted *P. olsonii* growth at 250 μL/L; thyme was effective for seven days; wild thyme and savory for two days. Thyme EO prevented *P. olsonii* spore germination at 500 μL/L for seven days, though germination occurred at half that concentration. The IC90 values varied between 256.2 and 138.7 μL/L depending on the strain. The vapor phase of EOs at 125 μL/L influenced the sensory characteristics of cherry tomatoes; however, for thyme and oregano, this effect was not negative due to their culinary association with tomato flavor. The selected EOs could be used to control and prevent postharvest fruit losses, but further research is needed to optimize their application.

## 1. Introduction

Fruits and vegetables are integral to a balanced, health-promoting diet due to their high content of bioactive compounds. Antioxidants, carotenoids, minerals, and a diverse array of micronutrients have been extensively linked to therapeutic and preventive effects against prevalent chronic conditions such as obesity, cardiovascular diseases, and diabetes [1]. In addition to their nutritional value and health benefits, fruit and vegetables contribute significantly to the economic development of their producing countries and are an important source of income [2]. Global fruit and vegetable production has experienced a significant expansion, demonstrated by a 30% increase in recent years [3]. Even though fruits and vegetables are among the most recommended health foods, they are also highly problematic in terms of safety and preservation [4]. These agricultural commodities often carry pathogenic microorganisms alongside their natural microbial content. Plant diseases and postharvest spoilage are responsible for significant losses to the world economy [5]. Fungal contamination reduces productivity during storage and contributes to postharvest losses of nearly 30% of the global food supply annually. Due to their composition, fruit and vegetables have very short shelf lives after harvesting. The respiration rate, ethylene production, storage conditions, and phenotypic, quantitative, and qualitative changes in fruit affect the acceptability and safety of these plant products [2,6,7,8,9]. In terms of fungal contamination, any stage of the supply chain from harvest to distribution is at risk [10,11]. The best known and most widespread phytopathogens are species of the genera *Penicillium*, *Aspergillus*, *Alternaria*, *Rhizopus,* or *Botrytis* [12]. Along with their negative impact on the food industry, the main health risks associated with micromycetes stem from their ability to produce secondary toxic metabolites, a characteristic particularly associated with the genus *Penicillium* [13].

*Penicillium* is a genus of ubiquitous saprophytic fungi, which are very undemanding of environmental and nutritional conditions. Some species of the genus *Penicillium* have a positive role in the decomposition of biological material, but from a food safety point of view, other species pose a risk of food spoilage [14]. This includes, for example, *P. roqueforti*, an alimentary fungus resistant to preservative acids that is often detected in cereals, cheeses, or baked goods [15]. *P. digitatum* (referred to as green mold) and *P. italicum* (blue mold) are commonly found on citrus fruits as causal agents of fruit rot [12]. The most studied species is *P. expansum*, a necrotrophic phytopathogen known for soft rot of fruit [16]. An almost unknown species in the category of postharvest pathogen is *P. olsonii*. It is characterized as a psychrotolerant and halotolerant species often found in soil and indoor air [17,18]. *P. olsonii* can contaminate strawberries, beans, and various other vegetables, causing blue rot. Recently, *P. olsonii* has been identified as a cause of postharvest rot in tomatoes in Pakistan, Serbia, and Canada. The infection in tomatoes is manifested by mottling, necrotic lesions, and fruit softening [19,20,21,22]. These findings are quite recent, as *P. olsonii* colonization is better known on animal products, for example, with frequent occurrences reported on fermented meats such as sausages [23,24].

Fungicides are used to manage postharvest contamination of plant products. However, growing consumer awareness regarding food safety and potential adverse effects of synthetic fungicides has resulted in greater scientific interest in natural fungicidal agents. This change, coupled with the increasing emergence of resistant microbial strains, has led to proposed European regulations aimed at restricting certain chemically active ingredients. These regulations seek to promote alternative control methods that are effective yet safer for health, the environment, and biodiversity. Consequently, substances of botanical and biochemical origin have become a significant area of research [6,9,25].

Essential oils (EOs) are secondary metabolites produced in the cytoplasm of plants either as part of their development or in response to stress and infection. Chemically, EOs are low molecular weight aromatic mixtures of a volatile and lipophilic nature, soluble in organic solvents. Extraction is mainly carried out by hydrodistillation and steam distillation. Their phytochemical profile includes compounds with different biological and physicochemical properties, mainly aromatic hydrocarbons, terpenes (monoterpenes and sesquiterpenes), terpenoids, and esters. Owing to their aromatic character and non-toxicity, they are used as fragrance ingredients in pharmaceutical products, but their demonstrated antimicrobial activity, biodegradability, availability, and cost-effectiveness present new opportunities for their application [26,27,28,29,30,31]. Lipophilic molecules tend to form micelles in the aqueous phase, limiting the effectiveness of liquid-phase EOs to bind to microbial cells. As a result, higher concentrations of EOs are needed, which may alter food’s sensory qualities. The volatile phase of EOs offers a way to preserve bioactivity at lower concentrations, making it a better option for fresh food preservation [32].

In this study, we hypothesized that EOs could extend fruit shelf life by influencing *P. olsonii* development without compromising the sensory quality of tomatoes. The objective was to quantify the antifungal efficacy of selected EOs on the growth of colony and spore germination of *P. olsonii*, followed by testing the most effective EOs in vivo on cherry tomatoes. The final step was a sensory analysis conducted to evaluate the impact of active EOs on cherry tomatoes.

## 2. Materials and Methods

### 2.1. Fungal Strains

The five tested strains of microorganisms were obtained by isolation from lesions of moldy cherry tomatoes purchased from supermarket chains in Slovakia. Four strains of *P. olsonii* were isolated from cherry tomatoes of Slovak origin (KMi-1026, KMi-1029, KMi-1030, and KMi-1031), and one strain (KMi-1028) was isolated from organic cherry tomatoes from Spain. Strains were identified at the species level by their morphological and physiological characteristics and molecular methods, and they are preserved in the Microorganism Collection of the Institute of Biotechnology, Faculty of Biotechnology and Food Science, Slovak University of Agriculture, Nitra. The fungal strains were cultured for five days at 25 ± 1 °C on Czapek agar with yeast extract (CYA, HiMedia™, Mumbai, India) for subsequent testing. Spore suspensions were adjusted to a concentration of 10^6^ spores/mL with saline enriched with Tween 80 (0.5%) (Lachema, Brno, Czech Republic). The concentration of fungal spores was determined using an EVE^TM^ automatic cell counter (NanoEnTek, Seoul, Republic of Korea) and verified with a Thoma cell counting chamber to ensure the consistency of all fungal isolates.

### 2.2. Fruits

Cherry tomato bunches were purchased from a local producer, Babindol Farm, s.r.o. (Babindol, Slovakia), Slovak origin, variety Tramezzino, I. quality class.

### 2.3. EOs

The research focused on testing ten commercially available plant EOs from the family Lamiaceae. The producers stated that the EOs were obtained by hydrodistillation. The EOs were stored at temperatures up to 5 °C. Gas chromatography coupled to mass spectrometry (GC-MS) was performed to characterize the components of the EOs, specifically an Agilent 7890B gas chromatograph coupled to an Agilent 5977A mass detector (Agilent Technologies Inc., Palo Alto, CA, USA), with a CombiPal 120 autosampler (CTC Analytics AG, Zwingen, Switzerland) and an HP-5 ms (30 m × 0.25 mm × 0.25 µm) capillary column (Agilent Technologies, Palo Alto, CA, USA) [14]. The methodology is explained in more detail in a previous study [33]. The identified and quantified components of the EOs are listed in Table 1.

### 2.4. Antifungal Activity of Volatile EOs In Vitro

In the first part of the trial, the potential inhibitory effect of EOs at the undiluted concentration was tested to select only effective EOs. The vapor diffusion method was used as follows: 9 cm Petri dishes containing 15 mL of CYA were centrally surface inoculated with 5 μL of a fungal suspension of *P. olsonii* spores at a density of 10^6^ mL/L, as described above. EO was applied in a volume of 50 μL to a 5 cm diameter filter paper (Whatman No. 1), which was then placed in the center on the lid of a petri dish. In this volume, the EO vapor corresponded to 625 μL of EO in one liter of air. All samples for each *P. olsonii* strain were tested in triplicate. In the control treatment, an equal volume (50 μL) of distilled sterilized water was applied to the filter paper. The Petri dishes were sealed with parafilm M to prevent vapor leakage and cultured upside down for two weeks. Colony counts were obtained on the 3rd, 4th, 7th, 9th, 11th, and 14th day of cultivation. The colony diameter was measured along two perpendicular axes using a diameter caliper. The inhibitory activity level of the EOs was calculated using the following relative inhibition (RI) equation, where c is the diameter of the fungal growth in the control (mm) and t is the diameter of treated colony growth.

RI = [(c − t)/c] × 100

### 2.5. Minimal Inhibitory Concentration Determination

The second step was to further specify the level of antifungal activity of the EOs once this activity was demonstrated in the first experiment at an initial concentration of 625 μL/L. The minimum inhibitory concentration (MIC) was determined by starting with a stock EO concentration of 500 μL/L, which was sequentially diluted using dimethyl sulfoxide (DMSO; Fisher, Belgium) to obtain final concentrations of 250, 125, 62.5, 31.25, and 15.625 μL/L. The methodology was the same as described in the previous chapter, with the exception that the diluted EOs were applied in 40 μL volumes and two three-section Petri plates were used for each variant. Each EO was prepared in six replicates. DMSO was used as a negative control in place of EO. The control served as confirmation of the strain’s viability. The MIC was defined as the lowest EO concentration at which no fungal growth was observed after 7 and 14 days of incubation.

### 2.6. EO Antifungal Activity on Cherry Tomatoes

EOs with the lowest MICs were tested under in vivo conditions. The inhibitory activity was tested on fresh cherry tomatoes in the immediate postharvest period. Tomatoes were sorted by size and weight to form unitary groups and disinfected by immersion in a 1% sodium hypochlorite solution for 60 s to eliminate surface microbial contamination. After rinsing with sterile distilled water and drying, they were wounded with a 1 mm puncture in the mesh. At the puncture point, the tomatoes were inoculated with 5 μL of *P. olsonii* suspension (10^6^ spores/mL) using a micropipette. The inoculated fruits were placed in sterile glass jars (volume 500 mL, diameter 115 mm, height 95 mm) with a circular closure sealed with a rubber liner (Bromioli Rocco, Fidenza, Italy). Filter paper (Whatman No. 1) enriched with EO was placed in the lids of the jars. Five EOs (thyme, oregano, savory, wild thyme, and spearmint) diluted with DMSO solution to two concentrations of 205 and 125 mL/L were tested. In the control, sterile distilled water was used instead of EO. For each variant, there were three replicates. The sealed jars were stored for 12 days in the dark at a room temperature of 21 ± 1 °C. Fungal growth was observed by the numbers of lesions within each sample on each day, except days 4 and 11. In addition to assessing the mycelial growth of the pathogen, we also observed the sporulation of *P. olsonii*, which was visibly detectable to the naked eye. In the table, the presence of sporulation is represented by an index number representing the number of punctures in which sporulation was visible.

### 2.7. Effect of EOs on Spore Germination

Seven-day cultures of strains KMi-1026, KMi-1029, and KMi-1030 were utilized to prepare a spore suspension with a concentration of 10^6^ spores per mL, as previously described. A 100 μL volume of *P. olsonii* suspension was inoculated uniformly across the surface of the culture medium (CYA). Filter paper discs (Whatman No. 1) were treated with 40 μL of thyme EO, diluted in DMSO to obtain concentrations of 500, 250, 125, and 62.5 μL/L. The samples were incubated for 7 days at 25 ± 1 °C, with observations recorded every 24 h. The control was treated with sterile distilled water. Three 1 × 1 cm sections of the medium containing the fungal culture were excised using a lancet, and the slides were stained to enhance visualization. An Olympus BX51 optical microscope (Olympus cellSens imaging software Ver. 4.3) was used for microscopy. Spore counting was performed in three fields of view, with a maximum of 100 spores per field. A spore was considered germinated if its length exceeded half of its original size. Two replicates were prepared for each day of measurement and were not used further after the readings were taken to maintain the concentration of active EO in the dish for each day.

### 2.8. Sensory Analysis

The selection of EOs for sensory analysis was based on the MIC at which the EOs remained effective. Cherry tomatoes were placed in 500 mL glass jars, and the gas diffusion method, as previously described, was employed. EOs from thyme, sage, savory, wild thyme, and spearmint were tested at a concentration of 125 μL/L. The experiment was conducted in duplicate, with the sample containers stored for 7 days at 21 ± 1 °C. Cherry tomatoes stored under the same conditions (21 ± 1 °C, 7 days) without the presence of EO were used as the control. Sensory evaluation was performed using a 9-point hedonic scale, where 1 = ‘unacceptable/very poor’ and 9 = ‘very good’. The cherry tomatoes were assessed for sensory attributes, namely, taste and acceptability of off-flavors, aroma and acceptability of off-aromas, appearance, aftertaste, and overall acceptability. The analysis was carried out by a panel of 10 trained individuals. Informed consent to participate was obtained from all subjects involved in the sensory analysis.

### 2.9. Statistical Analysis

The MIC results were statistically processed using probit analysis in R-Studio (RStudio 2023.06.0 Build 421) [34] for the resulting inhibitory concentrations IC50 and IC90. The effect of EOs on spore germination was also evaluated by probit analysis, conducted in Statgraphics Centurion XV (Statgraphics Technologies, Plains, VA, USA). The sensory analysis results were evaluated using one-way analysis of variance ANOVA (*p* < 0.05), with a subsequent post hoc Tukey HSD test.

## 3. Results

### 3.1. Antifungal Effect of EOs In Vitro

Out of the ten EOs tested at 625 μL/L, half exhibited complete inhibitory effects and were subsequently selected for further MIC determination, namely, EOs of thyme, oregano, wild thyme, savory, and marjoram. Three EOs demonstrated partial inhibition by the end of the cultivation period, with inhibition rates of 55.42%, 46.00%, and 32.62% for sage, basil, and rosemary EOs, respectively. Sage EO completely stagnated the growth of all strains by day 4 and retained over 50% efficacy by the end of cultivation (with the exception of one strain). Mint EOs did not achieve 100% inhibition across all strains. The inhibitory activity of bergamot mint EO decreased to 86.32% for two strains (KMi-1026 and KMi-1031) by the end of the cultivation period, while the effectiveness of spearmint EO diminished after four days, inhibiting the growth of strain KMi-1028 by 85.53%. Detailed results are presented in Table 2. For 100% effective EOs, MICs were determined in the next phase using all *P. olsonii* strains. Given the inhibitory potency of mint EOs (over 85%), further testing was conducted only on strains sensitive to these EOs to confirm or refute their antifungal effects at reduced concentrations.

### 3.2. Minimal Inhibitory Concentration

The MIC of thyme EO was determined to be 250 μL/L, at which it effectively inhibited all strains. It was sufficient to inactivate three strains (KMi-1029 to KMi-1031) even at a concentration twice as low (62.5 μL/L), the lowest concentration determined. Savory, wild thyme, and oregano EOs were fully effective at concentrations of 500 μL/L on both days 7 and 14. Savory EO was effective at 250 μL/L against four strains, while wild thyme was effective at 125 μL/L for one strain. Oregano EO was the second most effective, while marjoram EO was the least active, failing to fully inhibit growth even at 500 μL/L by day 14. *P. olsonii* strains showed variability when treated with mint EOs in previous experiments, so only strains that were completely inhibited at 625 μL/L were used for further testing. Bergamot mint EO was ineffective at lower concentrations, so we eliminated it from further testing. On the contrary, spearmint EO, similar to thyme, inhibited the growth of the four tested strains at 250 μL/L.

**Figure 1:**
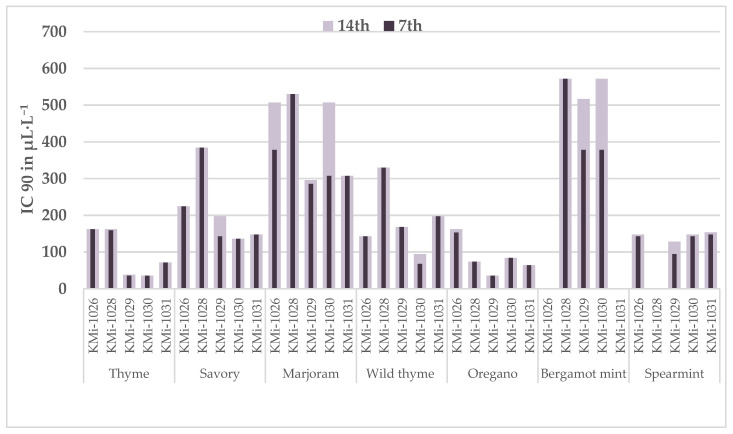

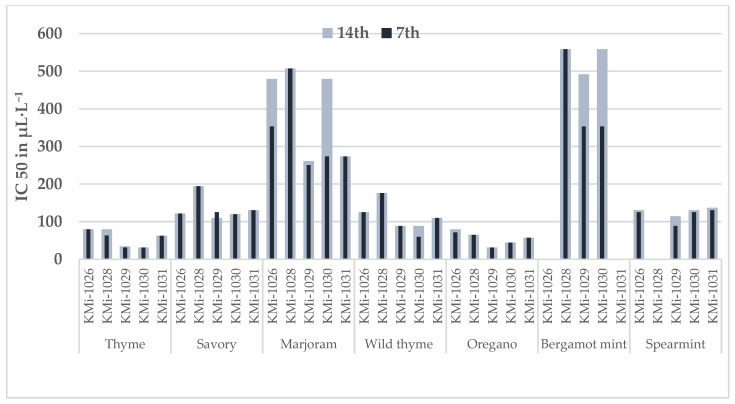
Estimated concentrations (μL/L) of EOs to inhibit 50% (IC50) and 90% (IC90) strains of *Penicillium olsonii,* determined by probit analysis.

Using probit analysis, we statistically determined the IC50 and IC90 values of the EO concentrations required to inhibit *P. olsonii* strains by 50% and 90%, respectively (Figure 1). The lowest IC90 values on day 14 were observed for thyme and oregano EOs, ranging from 35.72 to 162.72 μL/L. Oregano, wild thyme, and savory EOs demonstrated consistent efficacy, remaining stable regardless of cultivation time (except for one strain in each case). Marjoram EO was the least effective, with the highest IC90 values on day 14, ranging from 296.27 to 506.74 μL/L. Significant variability in the strain responses to the EOs was evident in the data. For instance, strain KMi-1030 was the most sensitive to thyme EO (IC90 35.72 μL/L) and the most resistant to marjoram EO (IC90 506.74 μL/L). The mean ratio between IC50 and IC90 was relatively high (spearmint, marjoram, and beragamot EOs in the range of 88–95%, for the others, the range was 67–73%). This indicated that EOs with lower ratios retained their potency even when the concentration decreased.

### 3.3. EO Antifungal Activity on Cherry Tomatoes

Cherry tomatoes deliberately contaminated with *P. olsonii* were used to assess the activity of selected EOs at 250 and 125 μL/L (Table 3). Oregano EO showed the strongest inhibitory effect among the tested EOs, completely inhibiting the growth of all strains at a concentration of 250 μL/L in every replicate. Oregano EO was the only treatment that showed partial inhibitory potential at day 12, even at a concentration of 125 μL/L. Pathogen growth was suppressed in some replicates, while all other EOs showed fungal growth in all replicates by day 12. Sporulation occurred in only one oregano EO-treated sample, and only after day 9. Thyme EO fully inhibited strain KMi-1029 at 250 μL/L. When used at 125 μL/L, rapid colony growth began after day 2, with sporulation observed. Wild thyme and savory EOs had a similar effect, inhibiting the pathogen for up to two days irrespective of concentration, after which they became ineffective on some strains. At the lower concentration of savory EO, all strains sporulated in at least 1 sample. Spearmint EO at 250 μL/L exhibited a fungistatic effect on all strains until day 9, while at 125 μL/L, it was effective only until day 3. All control samples showed growth on the first day of cultivation.

### 3.4. Effect of EOs on Spore Germination

**Figure 2:**
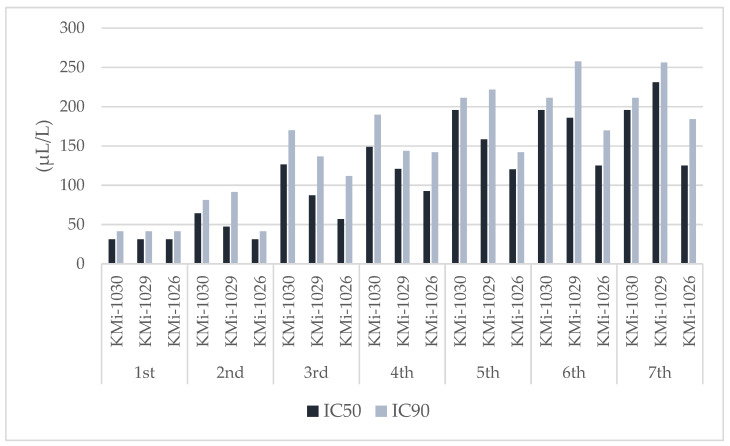
IC50 and IC90 (μL/L) of thyme EO for the inhibition of *Penicillium olsonii* spore germination.

Thyme EO completely inhibited spore germination in all strains for seven days at a concentration of 500 μL/L. At 250 μL/L, it was 100% effective against strains KMi-1030 and KMi-1026, while spores of KMi-1029 began germinating on day 5 in two replicates. At 125 μL/L, the EO inhibited KMi-1030 and KMi-1026 until day 2, with KMi-1026 showing sensitivity across three replicates until day 7. The resistance of KMi-1029 was again evident, as 250 μL/L inhibited germination for only one day. At the lowest concentration of 62.5 μL/L, germination was inhibited for two days in KMi-1029 and one day in the other strains. By the end of the cultivation period, 100% spore germination occurred in all replicates at concentrations of 125 μL/L and below, except for KMi-1029. To more accurately estimate the potency of thyme EO, the IC50 and IC90 values were determined using probit analysis (Figure 2). On day 1, the EO was consistently effective for all strains, with an IC90 of 41.34 μL/L. This concentration remained effective against KMi-1026 spores on day 2, while 141.89 μL/L was sufficient for days 4 and 5. For the other two strains, the required concentration for germination inactivation increased exponentially each day. The most sensitive strain was KMi-1029, with an IC90 of 256.15 μL/L by the end of the cultivation.

### 3.5. Sensory Analysis

**Figure 3:**
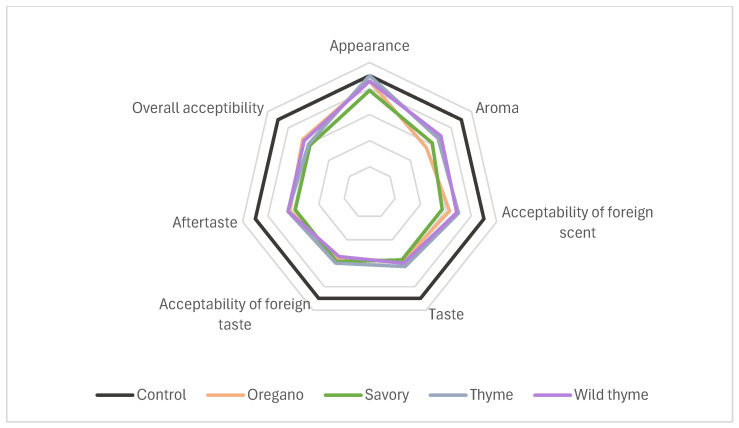
Radar plot of the sensory effect of EOs at a concentration of 125 μL/L on cherry tomatoes.

The four EOs with the lowest MICs determined in previous experiments were tested on cherry tomato sensory characteristics at a concentration of 125 μL/L for seven days and statistically processed using ANOVA. The control sample was predictably and correctly rated as the best. Only in the appearance trait was there no statistically significant differences between any pair of means at the 95.0% confidence level. Thyme EO was rated the same as the control. In all other evaluated traits, the samples differed to a certain extent from a statistical point of view (*p* < 0.05). In the aroma of cherry tomatoes, a significant difference was observed only for oregano EO, while in the acceptability of foreign aromas, savory EO stood out. Flavor-wise, all EO-treated samples differed from the control, with savory EO showing the greatest difference. The taste acceptability of oregano EO was rated the highest. The aftertaste of oregano, thyme, and wild thyme was more acceptable than that of savory. The panel attributed their positive ratings to the fact that herbs such as oregano and thyme are commonly used as flavoring agents, and thus their presence did not interfere with the perception of the tomatoes’ flavor. In terms of overall acceptability, cherry tomatoes carrying the sensory properties of oregano and wild thyme EOs were closest to those of normal cherry tomatoes (Figure 3).

## 4. Discussion

The cherry tomato is a widely cultivated fruit rich in vitamins but highly vulnerable to postharvest rot during processing and transport. The fungal pathogens *Botrytis cinerea* (grey mold) and *Alternaria alternata* (black rot) are the primary causes responsible for significant losses in cherry tomato production, causing severe spoilage and reducing the fruit’s marketability [35,36]. So far, only three occurrences of *P. olsonii* have been reported as the causal agent of cherry tomato rot in Pakistan [19], Serbia [21], and Canada [22]. According to Tančinová et al. [37], *P. olsonii* was the most common degradation factor on cherry tomatoes, identified in more than 57% of tested samples in Slovakia, and therefore we chose cherry tomatoes as a model commodity.

The diversity and potency of EOs make them a valuable subject of investigation for numerous studies. The antioxidant, antimicrobial, antiviral, and insecticidal activities of these compounds and their constituents have been repeatedly confirmed [38]. The bioactivity of EOs is determined by their chemical profile, suggesting that the response of pathogens to the effects of EOs may vary due to their different compositions. The inducers of biological effects are generally the main components (eugenol, citral, and cinnamaldehyde), which may be as effective alone as in a mixture of EOs; in other cases, minor constituents have been decisive in antimicrobial activity [39,40]. Vapor-phase EOs are reported to be particularly effective against fungi, as fungal growth involves airborne mycelium on agar surfaces, making it vulnerable to EO vapors. This study, therefore, focused on evaluating EOs as antifungal agents due to their effectiveness in the vapor phase [41].

This study aimed to further explore and clarify the antifungal effects of ten EOs known for their antimicrobial properties on the growth of 5 strains of *P. olsonii*, under both in vivo and in vitro conditions. In addition to the presence of micromycetes, indicated by the growth of white, cotton-like mycelium, we also observed sporulation. In *P. olsonii*, this was evident from the green, dotted surface coloration of the mycelium.

Based on the efficacy at the highest concentration (625 μL/L) used, the EOs could be divided into completely effective (thyme, oregano, wild thyme, savory, and marjoram EOs), more effective than ineffective (bergamot mint and spearmint EOs), and less effective (basil, rosemary, and sage EOs).

Basil is classified into four chemotypes due to the major constituents present in its EO: estragol, linalool, methyl eugenol, and methyl cinnamate. Considering the geographical origin of basil, the abundance of estragol in estragolic basil may be more than 80%, which was our case (84.89%) [42]. In the case of basil EO rich in oxygenated monoterpenes (linalool and 1,8-cineole), the EO showed antifungal activity against *Aspergillus* (*A. flavus*, *A. niger*, *A. teurreus*, and *A. carbonarius*) [43] and potent antistaphylococcal activity, similar to that of oregano, when applied at 1 mg/mL. The authors attributed the antibacterial activity to the major compounds of the EO: 65.2% linalool and 3.6% 1,8-cineole [44]. Even with almost equal proportions of phenolic and monoterpenoid compounds (49.94% estragole and 41.49% linalool), the vapor phase of basil EO significantly inhibited *Penicillium digitatum*; the minimum fungicidal dose was 300 μL, 350 μL for lavender EO, and peppermint EO had only a fungistatic effect [45,46]. In this study, basil EO was able to suppress the growth of only two strains of *P. olsonii* for three days. The lower activity of the EO may have been due to the composition with a predominance of estragole. Estragole showed limited antibacterial activity against *Shigella* spp. [47] and low activity was also observed for two strains of *Fusarium graminearum*; inhibitory activity was observed at MIC 12.5 mg/mL compared to MIC 0.4 mg/mL for thyme and oregano EOs [48].

According to several authors [49,50,51,52], the main compounds identified in rosemary EO universally include: 1,8-cineole (32.18%; 38.5; 4.90%; and 40.4%, respectively), camphor (16.20%; 17.1%; 41.22%; and 8.7%, respectively), α-pinene (15.40%; 12.3%; 17.49%; and 11.9%, respectively), and camphene (9.16%; 6.0%; 18.14%; and 3.5%, respectively). The effects of rosemary EO on the growth of *Alternaria alternata* and *Aspergillus niger* or antimicrobial effects on *Escherichia coli*, *Proteus vulgaris*, *Pseudomonas aeruginosa,* and *Listeria monocytogenes* have also been reported [49,50]. Corresponding to these results, the chemical composition of rosemary EO in our research is: eucalyptol 43.17% (1,8-cineole), α-pinene 10.74%, and (+)-2-bornanone 12.8% (camphor). However, rosemary EO was shown to have the weakest antifungal effect. The mean colony sizes of *P. olsonii* after 625 μL/L rosemary EO treatment ranged from 36.97 ± 0.29 to 52.49 ± 0.53 mm with an inhibitory efficiency of 32.62%. Hendel et al. [51] tested rosemary EO by fumigation against 32 foodborne pathogens involving 8 *Penicillium* species. At concentrations of 5, 10, and 15 μL/plate, the EO exerted more than 10% inhibition on 78.13, 87.50, and 96.88% of fungal isolates, respectively, while at a concentration of 15 μL/plate, it had an inhibition range of 50–100% on 21 isolates. Compared to thyme EO, rosemary EO appeared to be less effective.

Sage EO inhibited the growth of *P. olsonii* to 55.42% with a fungistatic effect up to day 4 of cultivation and colony sizes ranging from 19.89 ± 0.67 mm to 37.05 ± 0.38 mm. Sage EO has been reported to be less effective compared to thyme in in vivo and in vitro testing against *Colletotrichum acutatum*, a pathogen of strawberries. At a concentration of 1000 μL/L, the EO was active up to day 4; at 1800 μL/L, it had the highest potency of 88.14%, which, however, at the 7th day dropped to 62.54%. The growth of the pathogen on detached strawberry leaves was not affected (concentration of 1000 μL/L EO) [53]. Erarslan et al. [54] tested sage EO encapsulated in PVA/chitosan polymer against *A. niger* and *B. cinerea*. Encapsulated EO was more effective for controlling fungal mycelial growth than free EO, as there was a direct proportionality between the concentration and the effect of the EO. Among the different concentrations of sage EO, the solution of PVA/chitoate enriched with 1% EO was found to be the most effective; the MIC was determined to be 0.16 μL/mL. The major compounds identified in sage EO include caryophyllene (25.364%), camphene (14.139%), eucalyptol (13.902%), β-pinene (11.230%) [55], α, β-thujone (34.45%), camphor (20.46%), and eucalyptol (10.33%) [53]. In our study, we identified thujone (22.4%), camphor (19.7%), and eucalyptol (10.8%) in sage. One possible way to enhance the efficacy of EOs is to combine them. When 0.06% sage EO alone was applied, the mycelial growth area was about 15.9 cm^2^. When mixed with thyme EO in the same proportion (0.6%), the synergistic effect reduced mycelial growth to 0.1 cm^2^. This could be due to the enrichment of the mixture with the potent phytochemicals of thyme EO, γ-terpinene (68.415%) and p-thymol (24.721%) [55].

The complex species of genus *Mentha* is a globally widespread perennial herb, known for its medicinal and aromatic properties and for its important reserves of EOs [56]. Different *Mentha* species exhibit variations in the composition of their EOs, mainly due to differences in their specific metabolic pathways. The rate of biosynthesis is suggested to be the key determinant of monoterpene production, the main active component in these species [57]. *M. spicata* (spearmint) exhibits two main chemotypes: one with moderate-to-high carvone content and another rich in pulegone. The carvone-rich EO, containing up to 80% carvone along with limonene, terpinolene, and p-cymene-8-ol, has shown potential as a food preservative due to its antimicrobial and antiviral properties [56,57]. Differences in efficacy among *Mentha* species varieties are also shown in the study by Kowalczyk et al. [58]; the MIC percentage of the *M. spicata* ‘Nanah’ sample against *E. coli* was ≤0.098%, while *M. spicata* var. *crispa* ‘Persian’ had an MIC effect of 0.39%. Spearmint inhibited the growth of *Rhizopus stolonifer* mycelia by 92.41% after 72 h [59], as well as *Cryptococcus neoformans*, *Trichophyton rubrum,* and *T. verrucosum* (0.32 µL/mL) [60]. As mentioned above, the composition of EOs determines their activity. However, some components may also act antagonistically, which could explain the reduced activity of spearmint EO in our study. The main constituent carvone (ketone monoterpenoid) is known for its antifungal effects, whereas L-limonene as a hydrocarbon monoterpene has a limited ability to bind hydrogens, thus reducing not only its activity but also that of carvone. Carvone has low lipophilicity and hydrophobicity, is more difficult to bind, and alters membrane permeability [59]. Similarly, it is assumed that methyl acetate can reduce antifungal activity [60].

Spearmint ((−)-carvone 72.6%, D-limonene 15.2%) had 100% fungistatic effect on four strains of *P. olsonii* at a concentration of 625 μL/mL. Only in the case of KMi-1028 was its efficacy decreased to 85.86%, and the colony size was 9.94 ± 0.23 mm. Bergamot mint was less effective, inhibiting three strains of *P. olsonii* satisfactorily. Strain KMi-1031 had a colony diameter of 7.02 ± 0.26 mm on day 14, and KMi-1026 had a colony diameter of 12.02 ± 0.33 mm on day 11.

Since it was hypothesized that the strain response, rather than the intrinsic efficacy of mint EOs, influenced the antimicrobial results, we tested the oils at lower concentrations on sensitive strains to determine the MICs. For bergamot EO, this hypothesis was disproven, as it failed to inhibit mycelial growth at 500 μL/mL. However, spearmint EO confirmed the hypothesis with an MIC of 250 μL/mL, ranking third in effectiveness after thyme and oregano EOs. When tested in vivo on cherry tomatoes at the same concentration, spearmint EO showed a fungistatic effect by day 9, with sporulation observed in one sample. Differences in strain responses highlight the complexity of micromycetes and limit generalized claims of an EO effect on the fungus. A similar phenomenon was observed in the study by Valkova et al. [52], where rosemary EO showed no inhibitory effect on *P. crustosum* at concentrations of 125, 250, and 500 µL/L but inhibited *P. citrinum* and *P. expansum* at the highest concentration. However, when tested in vivo on bread, rosemary EO was the most effective, limiting the growth of *P. crustosum* by 93% at 250 µL/L, *P. expansum* by 86% at 125 µL/L, and *P. citrinum* by 57% at 500 µL/L.

In the MIC determination, marjoram EO failed to completely stop the growth of *P. olsonii* at the highest concentration used, 500 μL/L. The IC90 values estimated on day 14 ranged from 296.27 to 506.74 μL/L. Again, the different responses of the *P. olsonii* strains were confirmed: three strains were inhibited by IC90 > 500 μL/L, while on two strains (KMi-1029 and KMi-1031), the EO was sufficient at an IC90 around 300 μL/L. Similarly, for three strains (KMi-1028, KMi-1029, and KMi-1031), the IC90 was stable regardless of the day of cultivation. Since the antifungal ability of marjoram EO appeared to be limited, we did not test it in vivo on cherry tomatoes.

In the study by Jahani et al. [61], marjoram EO did not achieve complete inhibition (100%) of *Penicillium* spp., even at a concentration of 800 μL/L. When comparing the effectiveness of EOs against *Candida albicans* and *Aspergillus niger* (including basil, marjoram, clove, cumin, and caraway), marjoram EO was the least effective, with an MIC of 6 mg/mL for both microorganisms [62]. On the other hand, the two marjoram EOs tested at a concentration of 1 mg/mL effectively inhibited *A. alternata* mycelia by 90% and 74%, respectively [63]. Similarly, eleven isolates of *F. oxysporum* f. sp. melonis and ten isolates of *F. solani* were tested using the disc diffusion method in the presence of majorjam and lavender EOs. Both EOs had a significant effect on mycelial growth and completely inhibited spore germination. After application as a biofumigant, marjoram EO reduced the intensity of disease spread on melon by 23% after 20 days of cultivation (lavender by 60%) [64]. The marjoram EO chemical composition was as follows: terpinen-4-ol (34.94%), γ-terpinene (24.66%), α-terpinene (13.22%), β-terpinene (5.84%), α- terpineol (3.98%), and β-phellandrene (3.16%) [64]. Most studies agree that the main monoterpene alcohol component of marjoram EO is terpinene-4-ol [63]. This substance was also identified in our study in 34.% representation, together with γ-terpinene (16.9%) and *cis*-sabinene hydrate (15.1%).

Savory EO was fully effective at an MID of 500 μL/L on four strains at a concentration of 250 μL/L. The IC90 values ranged from 136.14 to 384.16 μL/L. KMi-1028 was more resistant than the others. The IC90 of savory EO determined at day 7 remained unchanged on day 14 (except for KMi-1092), which exhibited greater resistance over time. On cherry tomatoes, savory EO reduced the surface mycelium of *P. olsonii* by day 2 at both concentrations (250 and 125 μL/L), with sporulation beginning from day 5 at the lower concentration. These results are consistent with another study. In the in vitro contact phase, at concentrations of 10/20/30 μL per 20 mL, *S. hortensis* inhibited 100% of several *Penicillium* species (including *P. expansum* and *P. italicum*). In the vapor phase, the efficacy decreased in two cases to 92.06% at 10 μL and 88.06% at 20 μL. No inhibitory effect was observed on the growth of *P. digitatum* in vivo on lemon fruits [65]. Terpinene-4-ol, the main constituent of savory EO in our study, was reported to have only moderate antifungal activity against *Raffaelea quercus-mongolicae* and *Rhizoctonia solani* (>0.625 mg/paper disc), weak activity (0.313), and no activity at 0.156 mg/paper disc [66].

Wild thyme EO had similar activity to that of savory EO. The MID was determined to be 500 μL/L, with 125 μL/L being sufficient to inhibit one strain. The IC90 values estimated by probit analysis ranged from 94.61 μL/L to 330.21 μL/L. Similar to savory EO, strain KMi-1028 showed greater variability in resistance to the presence of the EO. Wild thyme EO has been confirmed to inhibit *Bacillus subtilis* biofilms [67] and supress the growth of *C. albicans* (MIC 0.039% to 0.078%) (31). Additionally, it showed potential as an antibiotic enhancer [68]. Wild thyme EO had only moderate inhibitory activity against *S. aureus* and *E. coli* (200 and 125 μL/mL, respectively) and a significant inhibitory effect against *C. albicans* (200, 125, and 62.5 μL/mL) [69].

Two of the most important and widely recognized aromatic plants for the extraction of EOs are oregano and thyme. The EOs derived from these plants exhibit a broad spectrum of biological activities, including antimicrobial, antimycotoxigenic, anti-inflammatory, antioxidant, and anticancer properties. Their bioactivity is largely attributed to their major active compounds, thymol and carvacrol, which are known for their potent effects. Regarding antifungal activity, these EOs have been extensively tested and demonstrated efficacy against a broad range of fungal pathogens, particularly species within the genera *Aspergillus*, *Penicillium*, *Fusarium*, and *Botrytis* [70,71,72]. These compounds are so potent that their relative concentrations within the overall composition directly correlate with the EOs’ efficacy. For instance, in tests evaluating the effectiveness of oregano EO against *Botrytis cinerea*, the EO had an EC50 of 52.92 mg/mL after three days. In comparison, thymol alone exhibited nearly three times greater potency with an EC50 of 17.56 mg/L, followed by carvacrol at 26.22 mg/L [73].

In our study, these findings were confirmed. Both oregano and thyme EOs exhibited comparable and significant antifungal activity against all strains of *P. olsonii*, as demonstrated in both in vitro and in vivo experiments. The MIC of thyme EO was 250 μL/L, while that of oregano was 500 μL/L. Thyme EO was the only one tested that exhibited partial efficacy even at the lowest concentration of 62.5 μL/L. For both EOs, the IC90 values were estimated to range from 35.72 to 162.72 μL/L. Oregano EO demonstrated complete antifungal activity in vivo on cherry tomatoes at a concentration of 250 μL/L. This MIC aligns closely with the antifungal activity observed for oregano EO in vitro; however, it inhibited only a single resistant strain, KMi-1026, at 500 μL/L. Oregano EO demonstrated a significant effect on mycelial growth and spore germination even at lower concentrations. In contrast, thyme EO’s efficacy decreased significantly compared to the in vitro assay. At 250 μL/L, thyme EO completely inhibited only one strain, exerting a fungistatic effect on additional strains up to day 7. At lower concentrations, fungal growth resumed after the second day, with sporulation observed by day 5.

The significant activity of these EOs can also be used to enhance the activity of other EOs. In a test against *F. oxysporum*, the MICs of oregano and thyme EOs were 0.156 μL/L and 0.313 μL/L, respectively, and combining the two together reduced the dose to 0.078 μL/L and 0.039 μL/L, respectively [71]. An enhanced antimicrobial effect against *Salmonella Typhimurium* and *Listeria monocytogenes* was also observed. [74]. It has been reported that mixtures of EO components (eugenol, carvone, and cuminaldehyde) in combination with plant EOs had a more significant antifungal effect due to their synergistic activity and reduced side effects against *C. albicans* and *A. niger* [62].

Thyme EO demonstrated significant inhibitory activity against *P. olsonii* spores over a 7-day cultivation period. It was effective at a concentration of 500 μL/L on all strains and at half concentration on two strains. The IC90 values were determined to range from 41.34 to 257.63 μL/L. The effect of oregano and thyme EOs on spore germination at a concentration of 0.078 μL/mL was also confirmed against *Fusarium* species. Thyme EO alone did not achieve 100% inhibition at this concentration [71]. In another study, oregano EO inhibited the germination of *Botrytis cinerea* after 48 h of incubation, with an MIC of 31.25 mg/L. However, thymol alone was more effective, fully inhibiting germination at a concentration of 7.81 mg/L [73]. Thyme EO and its major compound thymol showed a strong inhibitory effect on the germination process of *R. oryzae* sporangiospores (94 and 100%) by 24 h of incubation at concentrations of 512 and 256 µg/mL (EO) and 256 and 128 µg/mL (thymol), respectively [75].

Although the mechanism of action of EOs on fungal cells is still not fully understood, several pathways have been identified through which these compounds can disrupt and destroy fungal cells. In addition to inhibition of mycelial development and sporangiospore germination, direct interaction with ergosterol, the major sterol of fungi and yeasts, has also been demonstrated. This reaction leads to membrane disruption and leakage of internal cell contents [76]. In addition to limiting ergosterol production and reducing mycelial biomass, EOs may directly impact the biosynthesis of toxins, such as aflatoxins B1 and B2, even at concentrations below the MIC. This suggests that the antitoxigenic effect can occur independently of the antifungal activity [75]. Moreover, EOs (e.g., oregano) influence oxidoreductase activity, malondialdehyde production (lipid peroxidation), and sclerotia formation (shown in the case of *Rhizoctonia solani*) [77]. Another cause of fungal cell death may be the induction of reactive oxygen species (e.g., thymol and carvacrol) that disrupt fungal cell membranes [66].

The antifungal activity of EOs increases with concentration; however, at higher levels, the strong flavor can negatively impact the sensory qualities of products beyond the limits of acceptability. In the case of stored fresh fruit and vegetables, flavor and appearance deteriorate relatively quickly and are an indicator of shelf life for the consumer. An important criterion in the use of EOs regarding sensory characteristics is therefore the concentration and type of food targeted [2].

In our study, the control sample scored the best in all sensory traits of cherry tomatoes. The aim was to assess whether the treated samples differed from the control in terms of extraneous flavor and aroma, and whether these differences were acceptable. The samples had similar ratings, with cherry tomatoes stored in a savory EO-modified atmosphere receiving the lowest rating. There was a significant difference in the aroma trait between the control and the oregano sample. Nevertheless, oregano EO was rated the best in overall acceptability, along with motherwort and thyme. The ranking was justified by the fact that the flavors of some herbs are commonly used in the culinary industry as a food ingredient (pizza, puree, etc.).

The impact of EOs on the organoleptic properties of foods can be managed through encapsulation techniques. For instance, encapsulating peppermint EO in carboniferous wax has shown to be an effective approach for ice cream applications, as no significant differences in texture or color were observed in the treated samples [78]. Another way to reduce the undesirable effects of EOs on the sensory attributes of fruits is their application in coatings [79]. For example, 5% cinnamon EO suppressed the growth of *P. expansum* and preserved the flavor, freshness, and nutritional values of stored apples compared with uncoated apples, which were degraded toward the end of storage [80]. Another study found that pistachios coated with sodium alginate (1%) enriched with thyme EO (0.3% and 0.5%) received significantly higher sensory scores for certain evaluated attributes compared to the control samples [81].

The EOs with the most pronounced antifungal activity against all *P. olsonii* strains included oregano, thyme, motherwort, and savory. A recent study tested the effect of these 4 oils against *C. albicans* and *C. glabrata*, and the results were that thyme EO was the most effective (MIC 125–500 mg/L), followed by oregano and savory EOs with the same MIC of 250–500 mg/L. Wild thyme EO was the least effective (MIC = 500–1000 mg/L) [82]. Efficacy was again defined by their composition, the main constituents being carvacrol and thymol. The antifungal activity of monoterpenes of the phenolic group (thymol and carvacrol) is higher than that of hydrocarbons (α-pinene camphene, β-pinene, myrcene, α-terpene, p-cymene, limonene, and γ-terpene) or alcohols (terpinen-4-ol and linalool) [66]. In several studies [67,68], thymol was also identified as the major constituent, comprising 15.79% to 18.8% of the EO. However, in our investigation, thymol was the second most abundant constituent in wild thyme EO, accounting for only 12.13%. Although savory EO contained 20.2% thymol, it did not exhibit the expected level of efficacy. This underscores the significance of minor constituents in EOs and their potential antagonistic or synergistic interactions, which can markedly influence the overall bioactivity. In our study, oregano EO was identified as 60.37% thymol and thyme EO at 33.7%. Thus, it is consistent with both the results of this study and the facts regarding the efficacy of the components of EOs that thyme and oregano EOs were more effective. Although IC90 limits were set the same for both EOs; oregano EO was slightly more effective with an IC90 of 84.12 µL/mL (thyme IC90 94.02 µL/mL).

## 5. Conclusions

Thyme, oregano, wild thyme, marjoram, and savory EOs effectively demonstrated significant antifungal effects. All of them were able to inhibit mycelial growth under laboratory conditions and control *P. olsonii* development on cherry tomatoes. Thyme EO additionally impacted spore germination. The different responses of strains in the atmosphere of EOs highlight the importance of testing multiple strains within a single fungal species. EOs altered the sensory profile of the cherry tomatoes. However, thyme and oregano EOs were evaluated positively and have potential for application due to their well-known use as seasonings with tomato-based foods. Future research should focus on optimizing methodologies to minimize sensory changes while preserving the antifungal efficacy of EOs.

## Figures and Tables

**Table 1:** Quantitative analysis of chemical constituents in EOs exhibiting over 2% composition tested against *Penicillium olsonii*.

EO and Plant Source	Compound	Content in %
Thyme (*Thymus vulgaris* L.)	o-Xylene	43.9
	Thymol	33.7
	Linalool	7.1
	α-Pinene	3.5
Savory (*Satureja hortensis* L.)	γ-Terpinene	45.1
	Thymol	20.2
	p-Cymene	19.6
	(+)-4-Carene	3.8
Sage (*Salvia officinalis* L.)	Thujone	22.4
	(+)-2-Bornanone	19.7
	Eucalyptol	10.8
	Humulene	6.9
	β-Thujone	6.6
	α-Pinene	6.1
	Camphene	5.9
	Caryophyllene	5.6
	endo-Borneol	4.45
Spearmint (*Mentha spicata* L. var. *crispa*)	(-)-Carvone	72.6
	D-Limonene	15.2
Bergamot mint (*Mentha citrata* Erh.)	Linalyl acetate	45.0
	Linalool	34.0
	Geranyl acetate	5.9
Marjoram (*Origanum majorana* L.)	Terpinene-4-ol	34.5
	γ-Terpinene	16.9
	cis-Sabinene hydrate	15.1
	(+)-4-Carene	9.3
	Sabinene	6.9
	o-Cymene	6.3
Wild thyme (*Thymus serpyllum* L.)	Benzene, 4-ethyl-1,2-dimethyl-	18.07
	Thymol	12.13
	Geraniol	10.74
	γ-Terpinene	10.43
	Linalool	5.06
	Geranyl acetate	4.77
Oregano (*Origanum vulgare* L.)	Thymol	60.37
	Benzene, 4-ethyl-1,2-dimethyl-	13.14
	gamma-Terpinene	7.91
Basil (*Ocinum basilicum* L.)	Estragole	84.89
	Eucalyptol	4.1
Rosemary (*Rosmarinus officinalis* L.)	Eucalyptol	43.17
	(+)-2-Bornanone	12.8
	α-Pinene	10.74
	β-Pinene	7.43
	Camphene	4.66
	endo-Borneol	3.83
	Caryophyllene	3.78

**Table 2:** Partial inhibitory activity of basil, sage, rosemary, bergamot, and spearmint EOs on *Penicillium olsonii* (CYA, 25 ± 1 °C), expressed as growth diameter (diameter in mm, n = 3) and relative inhibition (%).

Strain	EOs	Av in mm	Day of Cultivation						
		RI (%)	2nd	3rd	4th	7th	9th	11th	14th
KMi-1026	Control	Av ± sd	10.26 ± 0.36	15.02 ± 0.34	22.01 ± 0.24	38.24 ± 0.65	47.54 ± 0.38	47.54 ± 0.38	72.95 ± 0.31
	Basil	Av ± sd	0.00 ± 0.00	0.00 ± 0.00	6.92 ± 0.16	12.78 ± 0.79	19.46 ± 0.37	24.24 ± 0.21	44.89 ± 0.36
		RI	100	100	90.51	82.48	73.32	66.77	38.46
	Rosemary	Av ± sd	0.00 ± 0.00	0.00 ± 0.00	7.04 ± 0.19	15.46 ± 0.50	22.72 ± 0.31	27.61 ± 0.43	49.19 ± 0.28
		RI	100	100	90.35	78.81	68.86	62.15	32.57
	Sage	Av ± sd	0.00 ± 0.00	0.00 ± 0.00	0.00 ± 0.00	10.46 ± 0.26	14.45 ± 0.22	18.59 ± 0.09	34.58 ± 0.65
		RI	100	100	100	85.66	80.19	74.52	52.60
	Bergamot mint	Av ± sd	0.00 ± 0.00	0.00 ± 0.00	0.00 ± 0.00	0.00 ± 0.00	0.00 ± 0.00	4.76 ± 0.26	12.02 ± 0.33
		RI	100	100	100	100	100	93.47	83.52
	Spearmint	Av ± sd	0.00 ± 0.00	0.00 ± 0.00	0.00 ± 0.00	0.00 ± 0.00	0.00 ± 0.00	0.00 ± 0.00	0.00 ± 0.00
		RI	100	100	100	100	100	100	100
KMi-1028	Control	Av ± sd	9.40 ± 0.13	13.22 ± 0.46	20.28 ± 0.19	32.74 ± 0.61	44.01 ± 0.91	54.38 ± 0.47	70.17 ± 0.27
	Basil	Av ± sd	4.64 ± 0.20	6.07 ± 0.20	8.42 ± 0.24	10.29 ± 0.21	13.29 ± 0.26	18.96 ± 0.29	32.42 ± 3.73
		RI	93.39	91.35	88	85.34	81.06	72.98	53.80
	Rosemary	Av ± sd	0.00 ± 0.00	0.00 ± 0.00	0.00 ± 0.00	8.65 ± 0.40	13.80 ± 0.20	18.50 ± 0.28	36.97 ± 0.29
		RI	100	100	100	87.67	80.33	73.64	47.31
	Sage	Av ± sd	0.00 ± 0.00	0.00 ± 0.00	0.00 ± 0.00	8.50 ± 0.43	11.84 ± 0.23	14.06 ± 0.40	27.23 ± 0.69
		RI	100	100	100	87.89	83.13	79.96	61.19
	Bergamot mint	Av ± sd	0.00 ± 0.00	0.00 ± 0.00	0.00 ± 0.00	0.00 ± 0.00	0.00 ± 0.00	0.00 ± 0.00	0.00 ± 0.00
		RI	100	100	100	100	100	100	100
	Spearmint	Av ± sd	0.00 ± 0.00	0.00 ± 0.00	0.00 ± 0.00	6.94 ± 0.18	7.40 ± 0.32	8.39 ± 0.19	9.94 ± 0.23
		RI	100	100	100	90.11	84.45	88.04	85.83
KMi-1029	Control	Av ± sd	10.11 ± 0.11	18.94 ± 0.62	21.72 ± 0.53	32.99 ± 0.80	43.40 ± 0.49	48.63 ± 0.42	61.28 ± 0.48
	Basil	Av ± sd	0.00 ± 0.00	0.00 ± 0.00	8.48 ± 0.71	11.06 ± 0.91	14.65 ± 0.63	18.62 ± 0.35	30.28 ± 0.68
		RI	100	100	86.16	81.95	76.09	69.61	50.59
	Rosemary	Av ± sd	0.00 ± 0.00	8.66 ± 0.13	14.96 ± 0.53	22.92 ± 0.37	27.01 ± 0.30	30.71 ± 0.41	45.07 ± 0.54
		RI	100	85.87	75.59	62.60	55.92	49.89	26.45
	Sage	Av ± sd	0.00 ± 0.00	0.00 ± 0.00	0.00 ± 0.00	11.12 ± 0.58	19.12 ± 0.62	22.08 ± 0.65	29.36 ± 0.65
		RI	100	100	100	81.85	68.80	63.97	52.09
	Bergamot mint	Av ± sd	0.00 ± 0.00	0.00 ± 0.00	0.00 ± 0.00	0.00 ± 0.00	0.00 ± 0.00	0.00 ± 0.00	0.00 ± 0.00
		RI	100	100	100	100	100	100	100
	Spearmint	Av ± sd	0.00 ± 0.00	0.00 ± 0.00	0.00 ± 0.00	0.00 ± 0.00	0.00 ± 0.00	0.00 ± 0.00	0.00 ± 0.00
		RI	100	100	100	100	100	100	100
KMi-1030	Control	Av ± sd	9.78 ± 0.40	17.49 ± 0.58	19.72 ± 0.62	33.68 ± 0.64	40.93 ± 1.11	48.80 ± 0.24	61.29 ± 0.73
	Basil	Av ± sd	0.00 ± 0.00	4.12 ± 0.11	4.90 ± 0.18	5.69 ± 0.56	7.79 ± 0.57	11.23 ± 0.28	21.49 ± 0.55
		RI	100	93.28	92	90.72	87.29	81.68	64.94
	Rosemary	Av ± sd	0.00 ± 0.00	0.00 ± 0.00	7.29 ± 0.12	20.92 ± 0.37	24.57 ± 0.39	29.01 ± 0.18	38.88 ± 0.45
		RI	100	100	88.11	65.87	59.91	52.67	36.56
	Sage	Av ± sd	0.00 ± 0.00	0.00 ± 0.00	0.00 ± 0.00	0.00 ± 0.00	9.09 ± 0.42	13.00 ± 0.25	19.89 ± 0.67
		RI	100	100	100	100	85.17	78.79	67.55
	Bergamot mint	Av ± sd	0.00 ± 0.00	0.00 ± 0.00	0.00 ± 0.00	0.00 ± 0.00	0.00 ± 0.00	0.00 ± 0.00	0.00 ± 0.00
		RI	100	100	100	100	100	100	100
	Spearmint	Av ± sd	0.00 ± 0.00	0.00 ± 0.00	0.00 ± 0.00	0.00 ± 0.00	0.00 ± 0.00	0.00 ± 0.00	0.00 ± 0.00
		RI	100	100	100	100	100	100	100
KMi-1031	Control	Av ± sd	11.64 ± 0.50	20.42 ± 0.45	26.94 ± 0.44	36.77 ± 0.28	44.16 ± 0.65	50.94 ± 0.60	65.79 ± 0.51
	Basil	Av ± sd	0.00 ± 0.00	7.17 ± 0.24	12.90 ± 0.44	20.41 ± 0.70	31.01 ± 0.35	36. 48 ± 0.60	51.21 ± 0.26
		RI	100	89.10	80.39	68.98	52.87	44.55	22.16
	Rosemary	Av ± sd	0.00 ± 0.00	10.01 ± 0.52	11.90 ± 0.29	20.19 ± 0.76	29.09 ± 0.24	36.94 ± 0.53	52.49 ± 0.53
		RI	100	84.78	81.91	69.31	55.78	43.85	20.22
	Sage	Av ± sd	0.00 ± 0.00	0.00 ± 0.00	0.00 ± 0.00	7.30 ± 0.54	18.53 ± 0.80	24.68 ± 0.29	37.05 ± 0.38
		RI	100	100	100	88.90	71.83	62.49	43.68
	Bergamot mint	Av ± sd	0.00 ± 0.00	0.00 ± 0.00	0.00 ± 0.00	0.00 ± 0.00	0.00 ± 0.00	0.00 ± 0.00	7.02 ± 0.26
		RI	100	100	100	100	100	100	89.11
	Spearmint	Av ± sd	0.00 ± 0.00	0.00 ± 0.00	0.00 ± 0.00	0.00 ± 0.00	0.00 ± 0.00	0.00 ± 0.00	0.00 ± 0.00
		RI	100	100	100	100	100	100	100

Legend: Av—mean, sd—standard deviation, CYA—Czapkov agar with yeast extract.

**Table 3:** Growth and sporulation of *Penicillium olsonii* strains in the presence of EOs in vivo on cherry tomatoes (21 ± 1 °C).

EOs	Day	*Penicillium olsonii* Strains					
		1026		1029		1030	
		Concentration of EOs (μL/L)					
		250	125	250	125	250	125
Thyme	1.	0/9 *	0/9	0/9	0/9	0/9	0/9
	2.	0/9	0/9	0/9	0/9	0/9	0/9
	3.	0/9	2/9	0/9	1/9	0/9	0/9
	5.	0/9	7^2^/9 **	0/9	4/9	0/9	4/9
	6.	0/9	9^2^/9	0/9	5/9	0/9	7/9
	7.	0/9	9^2^/9	0/9	7/9	0/9	7/9
	8.	1/9	9^2^/9	0/9	8/9	0/9	8/9
	9.	1/9	9^2^/9	0/9	9/9	0/9	8/9
	10.	1/9	9^2^/9	0/9	9^1^/9	1/9	9/9
	12.	1/9	9^2^/9	0/9	9^1^/9	2/9	9/9
Oregano	1.	0/9	0/9	0/9	0/9	0/9	0/9
	2.	0/9	0/9	0/9	0/9	0/9	0/9
	3.	0/9	2/9	0/9	0/9	0/9	0/9
	5.	0/9	3/9	0/9	0/9	0/9	2/9
	6.	0/9	5/9	0/9	2/9	0/9	3/9
	7.	0/9	7/9	0/9	2/9	0/9	4/9
	8.	0/9	7/9	0/9	6/9	0/9	5/9
	9.	0/9	7^1^/9	0/9	6/9	0/9	5/9
	10.	0/9	7^1^/9	0/9	6/9	0/9	5/9
	12.	0/9	7^1^/9	0/9	7/9	0/9	5/9
Wild thyme	1.	0/9	0/9	0/9	0/9	0/9	0/9
	2.	0/9	0/9	0/9	0/9	0/9	0/9
	3.	0/9	1/9	0/9	0/9	1/9	0/9
	5.	4/9	5/9	0/9	3/9	4/9	2/9
	6.	6/9	7^1^/9	0/9	5/9	6/9	3/9
	7.	9/9	8^1^/9	0/9	8/9	7/9	5^1^/9
	8.	9/9	8^3^/9	2/9	9/9	7/9	8^3^/9
	9.	9/9	8^3^/9	2/9	9/9	8/9	9^3^/9
	10.	9/9	8^3^/9	2/9	9/9	8/9	9^3^/9
	12.	9/9	9^3^/9	2/9	9/9	8/9	9^3^/9
Savory	1.	0/9	0/9	0/9	0/9	0/9	0/9
	2.	0/9	0/9	0/9	0/9	0/9	0/9
	3.	1/9	4/9	0/9	0/9	0/9	0/9
	5.	3/9	8^2^/9	2/9	6/9	6/9	6^2^/9
	6.	5/9	8^2^/9	4/9	8^2^/9	6/9	7^3^/9
	7.	6/9	8^3^/9	5/9	9^3^/9	8/9	8^3^/9
	8.	6/9	8^4^/9	5/9	9^5^/9	8/9	9^3^/9
	9.	6/9	8^4^/9	6/9	9^5^/9	8/9	9^3^/9
	10.	6/9	9^4^/9	6/9	9^5^/9	8/9	9^3^/9
	12.	6/9	9^4^/9	7/9	9^5^/9	8/9	9^3^/9
Spearmint	1.	0/9	0/9	0/9	0/9	0/9	0/9
	2.	0/9	0/9	0/9	0/9	0/9	0/9
	3.	0/9	0/9	0/9	0/9	0/9	0/9
	5.	0/9	6/9	0/9	2/9	0/9	1/9
	6.	0/9	9^4^/9	0/9	7/9	0/9	8/9
	7.	0/9	9^5^/9	0/9	8/9	0/9	9^1^/9
	8.	0/9	9^7^/9	0/9	9^1^/9	0/9	9^4^/9
	9.	0/9	9^8^/9	0/9	9^1^/9	0/9	9^7^/9
	10.	1/9	9^8^/9	2/9	9^1^/9	2/9	9^7^/9
	12.	4/9	9^8^/9	2/9	9^1^/9	3/9	9^7^/9
Control	1.	7/9		5/9		4/9	
	2.	9/9		9/9		6^1^/9	
	3.	9^9^/9		9/9		9^1^/9	
	5.	9^9^/9		9^8^/9		9^8^/9	
	6.–12.	9^9^/9		9^9^/9		9^9^/9	

* number of *P. olsonii* lesions in 9 inoculated cherry tomato points. ** superscript—number of sporulating colonies.

## Data Availability

The original contributions presented in the study are included in the article, further inquiries can be directed to the corresponding author.
